# Optimal starting point for antiretroviral HIV treatment in a town in Cameroon: a randomised controlled study

**DOI:** 10.1186/1471-2458-14-828

**Published:** 2014-08-10

**Authors:** Knut Holtedahl, Daniel Salpou, Tonje Braaten, Zogoi Berved

**Affiliations:** Department of Community Medicine, UiT The Arctic University of Norway, Tromsø, Norway; Protestant Hospital, Ngaoundere, Cameroon

## Abstract

**Background:**

Optimal starting point for antiretroviral treatment (ART) has been uncertain.

**Methods:**

Parallel group, single blind, randomised controlled study of adult HIV positive patients consulting at the Protestant Hospital, Ngaoundere, Cameroon in 2007-8. Simple randomisation of patients in WHO clinical stage 1-2 to start of ART early or deferred, i.e. when CD4 counts dropped below 350 versus 250 cells/mm^3^, or when they reached clinical stage 3-4. Clinical follow-up every three months were offered for all patients. Main outcomes were clinical stage, CD4 differences and mortality. Of 424 consulting patients, most were excluded, mainly because they were already in WHO stage 3-4. Forty-four patients were randomised.

**Results:**

In the ‘early’ group two patients died and five were lost to follow-up. In the ‘deferred’ group, six patients died and nine were lost to follow-up (Hazard ratio for death by early compared to deferred treatment 0.26, 95% confidence interval 0.05-1.29). Of the patients lost to follow-up, three patients in the ‘early’ group and four patients in the ‘deferred’ group were known to be alive when the study ended. Fourteen patients in the early group and 11 in the deferred group started ART. Twenty-two patients were evaluated clinically six to seven months after the study period was terminated. Except for one patient with AIDS, these were all still in clinical stage 1-2.

**Conclusions:**

In our small sample, relative risk for death did not differ significantly, but deferred treatment seemed to carry no increased survival or other clinical advantage. During the study period, other studies made WHO change its guidelines to conform to our early treatment. The tendency in our study lends support to this policy.

**Trial registration:**

ISRCTN22114173

**Electronic supplementary material:**

The online version of this article (doi:10.1186/1471-2458-14-828) contains supplementary material, which is available to authorized users.

## Background

With the 2010 and 2013 guidelines, WHO recommended earlier initiation of antiretroviral treatment (ART) than in previous guidelines [1]. Evidence for a beneficial effect of earlier start of treatment was shown first in Western countries [2–4]. Later on, studies from African countries and Haiti have supported this [5–8]. Population effects pointed in the same direction [9–11]. Randomised studies are few and there has recently been a call for more research about when to start ART in Africa [12]. One reason we started our study in 2007, was precisely because our clinical impression from around 2003 was that ART was initiated too late. Other authors had argued that WHO patients with clinical stage 3 and 4 should initiate treatment [13]. ART became accessible in Cameroon around 2002-4, both in terms of drug supply and through a sharp decline in prices, ending with free distribution of some drugs in large parts of the country from around 2005. CD4 counts have been accessible more or less continuously from 2005, but are usually free of charge only during campaigns and for pregnant women. Recommendations for low income settings before 2010 were to initiate treatment when a patient’s CD4 count dropped below 200 cells/mm^3^. National guidelines were in agreement with this. We report results from our study, which to some extent answered the original question of when to start, but also revealed some unexpected experiences.

## Methods

All new cases of adult treatment-naïve HIV-positive patients diagnosed between 15 March 2007 and 31 December 2008 at the Protestant Hospital in Ngaoundere, a town of 300 000 inhabitants in Northern Cameroon, were considered eligible for the study. They were randomised to initiating treatment with CD4 counts of either <250 or <350 cells/mm^3^, or when their condition after randomisation progressed to WHO clinical stage 3 or 4. Exclusion criteria were:

1. Patient fulfilled current local indications for immediate treatment: CD4 < 250 cells/mm^3^ or WHO clinical stage 3 or 4.
2. CD4 was currently >450 cells/mm^3^. This was because the patient would probably not start ART during the first year.
3. Age less than 16 years.

Enrolled patients who did not start treatment right after randomisation were scheduled to have clinical and CD4 controls free of charge every three months. After start of treatment, control of CD4 was offered every six months. Outcome measures were WHO clinical stage, mortality, changes in CD4 and weight maintenance. Because patients were to be recruited in WHO stage I-II, we did not expect many deaths during the three and a half years our study was meant to last. Power calculations therefore were made based on substitute outcomes, i.e. differences in cd4 values and weight loss. A mean group difference in final CD4 value of 100 cells/mm^3^ and a mean weight loss difference of two kilos were considered clinically important. In our calculations we used anticipated cd4 values of 400 and 300 cells/mm^3^ and a standard deviation of 100, and for weight loss 5 ± 2 kg and 3 ± 1.5 kg. To show such differences, calculations with significance level 5% and a statistical power 90% in both cases suggested that 21 patients in each group were needed [14].

The choice of drugs for ART in Ngaoundere was very limited. In practice almost all patients in 2007-8 received a one drug combination of lamivudine, stavudine and nevirapine. The exception was patients previously treated for Tb, who received efivarenz instead of nevirapine; three patients in our study. From 2008-9 serious stavudine side effects caused a gradual change of the three-drug pill to one where stavudine was replaced by zidovudine. This was the same for both groups. Cotrimoxazole was given routinely to all patients receiving ART, and also to other patients coming for routine follow-up.

Patients who started ART were informed about the reason for this, either cd4 level, WHO clinical stage, or both.

The study was scheduled to follow patients at least until the end of 2010. We terminated our study in July 2010, six months before planned, because both the WHO and the Cameroonian recommendations had changed during the preceding months, and the changes were in accordance with our early start of treatment. We then actively searched for the deferred therapy patients who did not present themselves according to their follow-up plan. Postal street addresses do not exist in Ngaoundere, but some of the patients could be contacted by mobile phone. During the first months of 2011 we were able to carry out the planned independent clinical evaluation for half of the original patients. It took some time to find the whereabouts of all patients, and censoring as dead, lost-to-follow-up or having been clinically evaluated was done with 30 June 2011 as the ultimate date.

### Randomisation and masking

The patients gave written, informed consent after clinical examination by a medical doctor. Printed information in French, Fulfulde and Haoussa (English translation, Additional file 1) was prepared and either read by the patient or read aloud in the preferred language when the patient was illiterate. After consent, randomisation was performed by opening the top envelope in a stack containing numbers prepared from a table of consecutive random numbers. Randomisation was thus open to the study group, but blinded for the patients. They were told before signing that they would receive ART when the follow-up showed it was time to start treatment.

The final evaluation in January-February 2011 was performed by one of the authors (ZB), a specialist in infectious diseases, who had recently been employed by the hospital and had no previous knowledge of the patients. Thus, he was blinded to each patient’s group belonging.

### Statistics

The Kaplan-Meier plot was applied to show the estimated survival functions for the two treatment groups. We used the Cox Proportional Hazards Model to calculate the hazard ratio for death according to treatment, with a corresponding 95% confidence interval. Intention-to-treat analyses were performed.

### Role of the funding source

The authors designed and performed the study without any interference from the two funding sources.

## Results

**Figure 1:**
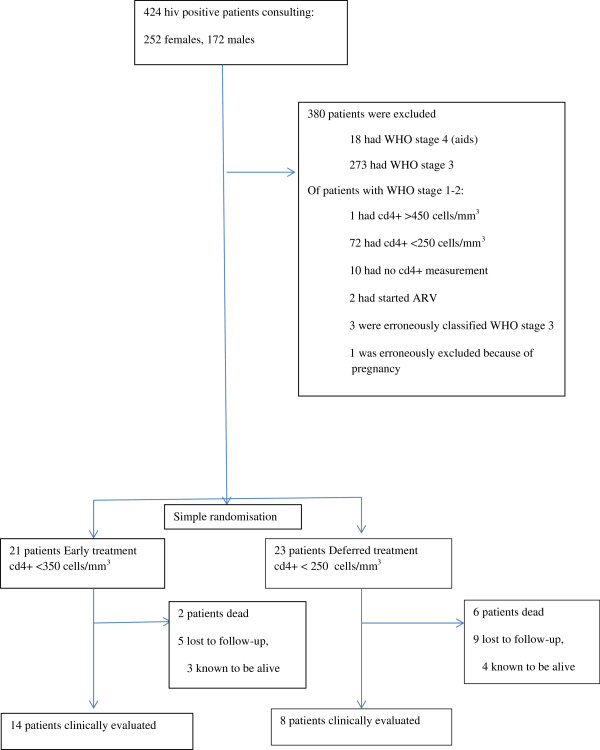
**Exclusions, inclusions and evaluation.**

Between April 2007 and November 2008, 424 HIV positive patients consulted at the Protestant Hospital. Most patients fulfilled one or more of the exclusion criteria. They therefore did not go through the randomisation procedure, and most of them started ART within a few weeks. CD4 was measured in 381 patients. Mean values were 174 cells/mm^3^ for 227 females and 138 cells/mm^3^ for 154 males. Forty-four patients were enrolled in the study, 28 females and 16 males, with a mean age of 31 and 36 years, respectively. Five patients were pregnant when they were diagnosed and recruited into the study, they did not previously know they had seroconverted and were ART-naïve like the other patients. Baseline characteristics are shown in Table 1. Exclusions are shown in Figure 1. During follow-up, patients had from zero to seven visits with CD4 count, more regularly after initiated ART therapy because this usually meant monthly visits with renewal of drug supply.

**Table 1:** **Baseline data, randomised patients (N = 44)**

Characteristic	Early treatment cd4+ <350 cells/mm3 (N = 21)	Delayed treatment cd4 + < 250 cells/mm3 (N = 23)
Age in years, mean (median)	33 (30)	33 (32)
Females	12	16
Males	9	7
Education (N = 41)		
No schooling	5	7
1-4 years of school	3	9
5-8 years of school	6	2
9+ years of education	6	3
Living with partner(s) or alone (N = 42)		
With partner	15	9
Alone	5	13
Pregnant	1	4
WHO stage 1	15	13
WHO stage 2	6	10
Pulmonary tbc, unknown when randomised	0	2
Cd4+ count, cells/mm3, mean (median)	351 (360)	340 (337)
Body mass index, mean (N = 7 + 5)	22 · 9	20 · 4
Weight in kilos, mean (N = 8 + 8)	63 · 2	57 · 8
Hemoglobin concentration, g/dl, mean (N = 14 + 18)	13 · 2	12 · 2

**Figure 2:**
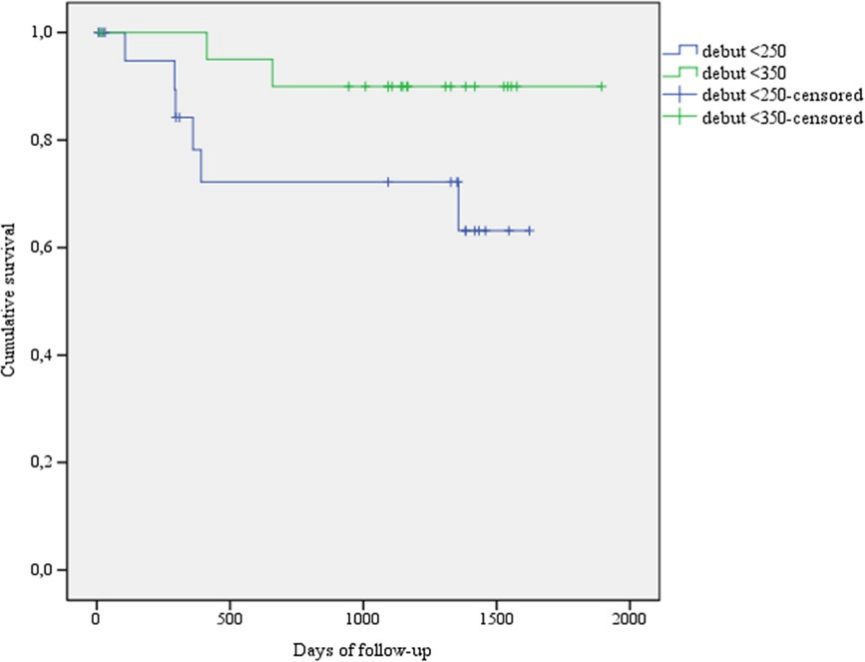
**Kaplan-Meier plot with estimated survival functions for the two treatment groups.**

Fourteen patients randomised to early treatment started ART, 13 because of CD4 < 350 cells/mm^3^, the last patient erroneously because an attack of herpes zoster was considered as stage 3. This patient later died. Eleven patients randomized to deferred treatment started ART, seven of them because they went below the threshold of <250 cells/mm^3^, four because they reached WHO stage 3. Two females randomised to early treatment, and four females and two males randomised to deferred treatment, died. Of the eight dead patients, one ‘early’ and three ’deferred’ patients had not started ART when they died 3 to13 months after randomisation. The other four died from 9-39 months after randomisation and 3-39 months after ART had been started. Figure 2 suggests an improved survival in the early treatment group, although not significant in such a small sample (HR = 0.26, 95% CI 0.05-1.29). Twenty-two patients went through the planned clinical evaluation at the end of the trial. One patient in the deferred group had AIDS, one in the early group was in WHO stage 2, the twenty others were asymptomatic (Table 2). Mean CD4 in the ‘early’ group was 556 and in the ‘deferred’ group 382 cells/mm^3^, this difference is not significant.

**Table 2:** **Outcome data, randomised patients (N = 44)**

Outcome	Early treatment cd4+ <350 cells/mm3 (N = 21)	Delayed treatment cd4 + < 250 cells/mm3 (N = 23)
Dead	2	6
Lost to follow-up	5	9
-but known to be alive when project ended	3	4
Started ARV	14	11
Alive with ARV	12	8
Number of days from diagnosis to start of ARV, mean (median) (N = 12 + 8)	390 (164)	340 (276)
Not started ARV	7	12
Alive without having started ARV	4	3
Number of days from diagnosis to censoring, mean (median)	857 (808)	585 (562)
Evaluated patients (N = 22)		
Number of patients evaluated (Females + Males)	14 (8 + 6)	8 (6 + 2)
Age in years, mean (median)	35 (32)	39 (40)
WHO clinical stage		
stage 1	13	7
stage 2	1	-
stage 4 (aids)	-	1
cd4+ count, cells/mm3, mean (median) (N = 14 + 7)	556 (547)	382 (319)
Body mass index (BMI), mean (N = 14 + 8)	23 · 6	22 · 8
Weight in kilos, mean (N = 14 + 8)	65 · 2	62 · 6
Haemoglobin concentration, g/dl, mean (N = 14 + 7)	12 · 7	11 · 2

Of the 14 patients lost to follow-up, three patients randomised to early treatment and one to deferred treatment moved to other towns, but some had occasional telephone contact with the hospital. Patients from distant locations were offered travel money if they would come to the final evaluation, but only one came. Vain efforts were also made to have patients who had moved inform us of their CD4 values taken elsewhere. Six of the eight dead patients never had any CD4 follow-up, and so was the case for three "early" and four "deferred" patients lost to follow-up. The average BMI for the evaluated patients was similar for the two groups. Of the dead patients, two had no schooling, four had 1-4 years and two had 5-8 years of schooling.

## Discussion and conclusions

Our study had a similar design as the Haitian study by Severe et al [6], who started their study in 2005 and had AIDS as their clinical exclusion criterion. When our study started two years later, the CD4 criteria in Cameroon followed WHO and national guidelines, with ART-start when CD4 dropped below 200 cells/mm^3^. However, it had become common to start treatment for patients in WHO clinical stage III. At that time in Cameroon, it therefore seemed unethical to expose stage III patients to deferred treatment, and it turned out that a majority of potential study patients had to be excluded because of this. With few patients and incomplete follow-up, our results are much less clear than the Haiti study, but their tendency is the same. Deferred treatment seemed to carry no increased survival or other clinical advantage. Sensitivity analyses were performed where all patients lost to follow-up and not known to be alive were counted as dead, and with all patients lost to follow-up counted as alive, but these analyses did not change conclusions (HR 0.42 (95% CI 0.15-1.22), and HR 0.33 (95% CI 0.07-1.66)), respectively. Counting the evaluated patient with AIDS among the dead gave only minimal changes. With few studies adding to current evidence, we think our study tells something about existing practical possibilities of HIV therapy and follow-up, which are probably common to many places in Africa.

It is not surprising that a majority of the patients who came for evaluation were among the least sick, but it is striking and encouraging that almost all were asymptomatic. This is in agreement with our clinical impression that patients who come regularly for their ART drugs in most cases improve clinically and remain well. Also, patients coming regularly to controls and who have a relatively high CD4 value usually avoid serious clinical deterioration before ART can be started. This is in spite of the limited local therapeutic offer of one standard ART combination and one available second line alternative. In Cameroon, scale-up of ART has been shown to be associated with increased drug resistance mutations [15], and resistance monitoring may be the next priority after CD4 access [16]. Routine measuring of viral load seems to be a lesser priority [17, 18]. In 2013, consolidated WHO guidelines have recommended early start of ART on an even broader base [1].

Median CD4 and BMI were slightly lower at baseline for the deferred group, which had relatively more females. The difference may have contributed to the higher number of dead or lost to follow-up, but we think the deferred treatment was more important. It may be worth noting that all the dead patients had little or no schooling.

To get an idea about natural CD4 decline without treatment, and thus about the lead time bias for the deferred treatment patients, we had foreseen an auxiliary study of patients who had at least one repeat measure of CD4 before ART became common. For this purpose, we went through hand-written medical records for all patients during the years 2003-7 at an HIV clinic in the region. We found 154 patients with repeat measures before any written signs of prescriptions of ART drugs. However, many of these patients had unexplained increases of CD4, which suggested that they had been treated in spite of no mention of this in the medical record, and we have not used these data. Without such data and also missing CD4 data for several patients, differences in mean CD4 values in the two groups are difficult to interpret. In the Ivory Coast it has been shown that 75% of ART-naïve patients had values of less than 350 cells/mm^3^ within seven years after seroconversion [19].

### Strength and limitations of the study

The randomised design is a strong element in our study. There are several weak points, mainly the low number of patients and the incomplete follow-up, resulting in unclear answers to our main questions. The setting is realistic and probably not very different from many African hospitals in towns and villages. The hospital is relatively well staffed with competent doctors and skilled nurses.

### Comparison with existing literature

While we were performing our study, other authors added to the evidence that earlier ART start might be beneficial [20, 21], both for survival [3] and for reducing morbidity and transmission [9]. Loss to follow-up was a problem in our study, and has been shown to be most important between HIV testing and CD4 testing [22, 23]. Delayed first consultation after diagnosis was reduced through decentralized activities in a Cameroonian study [24]. A large study from Kenya, Uganda and Zambia confirmed that many aspects of ART care could potentially be carried out safely and effectively at smaller, lower complexity facilities with less-specialised personnel [25]. Our patients had to wait for their CD4 results, and point-of-care cell counts have been shown to reduce loss to follow-up in some settings [26]. When comparing cost-effectiveness of CD4 cell count and WHO clinical staging to guide initiation of ART, CD4 cell count was superior in a study with data from sub-Saharan Africa [27] and in a study from Uganda [28].

Risk of HIV infection in Africa has been shown to be associated with a low educational level [29], and more than half of our patients had less than five years of schooling.

### Implications for clinical practice and research

Patients with HIV may benefit from ART while they are still asymptomatic. Cytometric CD4 testing is a good and cheap method for evaluating optimal starting point for ART, and more studies are needed to further document recommended thresholds in WHO guidelines [5]. In resource-limited settings, competent HIV services need to be decentralised to make appropriate follow-up available in a realistic way. General education is a necessity in modern society, among other things to help people understand how to avoid the virus and how to get follow-up if seroconversion occurs. Large, well-financed research projects often give the best answers to how to treat HIV, but smaller local clinical studies are also needed to increase the evidence base and implement new knowledge.

## Ethical approval

The study was cleared by The Regional Committee for Medical Research Ethics in Western Norway (REK III nr 115.02), and permission was given by the Provincial Head of medical services in Adamawa province where Ngaoundere is located. The study was reported to the Norwegian Social Science data services, project no 9248/02. The study was web-registered at ISRCTN22114173 http://www.controlled-trials.com/isrctn/search.html?srch=22114173&sort=3&dir=desc&max=10&Submit=SUBMIT. The CONSORT checklist has been used for the abstract.

## Funding

Funding was modest, approximately 9-10000 Euro a year between 2006 and 2012, mainly used to pay the laboratory technician who has been a stable clinical first line health worker throughout the study. Part of the money covered the scheduled follow-up consultations, including local transport and blood tests, sometimes also for non-ART drugs not covered otherwise. Travel between Cameroon and Norway has been covered for the author KH.

## Electronic supplementary material

Additional file 1: **Patient information, English translation.** (DOC 25 KB)
