# Supplementary material for: Optimal starting point for antiretroviral HIV treatment in a town in Cameroon: a randomised controlled study
Source: BMC Public Health. 2014 Aug 10;14:828. doi: 10.1186/1471-2458-14-828 (PMC4138380; doi:10.1186/1471-2458-14-828)
Supplement: Supplementary file 1 — Additional file 1: Patient information, English translation. (DOC 25 KB) [file 12889_2013_6954_MOESM1_ESM.doc]

**Written and oral information to patients**

-to be read aloud in one of the three major languages, French, Fulfulde (understood by almost everybody) or Hausa. A written copy in French or Fulfulde will be given to the patient, regardless of ability to read and write.

As a person living with the hiv virus, you have now been accepted by the provincial committee for treatment when the time comes. You have been informed that your latest blood test shows that the time for treatment may come during the next months or years. The best indicator for this is the counting of a certain type of cells or particles in your blood. The treatment aims at keeping your disease in check so that you will stay healthy even if the virus cannot be taken away from your body.

With this virus, much is known and much is unknown. One of the things we do not know for sure, is when is the right time to start treatment. We therefore ask you whether you will participate in a research project which *aims at finding the best time for starting treatment*.

Participation is voluntary, and you may withdraw from the project at any time. Treatment will continue as usual in case you withdraw from the study. If you participate, you will be assigned haphazardly to one of two groups which will start treatment on the basis of two different concentrations of blood cells. We cannot to-day say which level is the best to start at, this is what we hope to clarify with your help and participation.

All information in your medical record is confidential, and so is all additional information related to this project. The study is a collaboration between your local hospital and the University of Tromsø in Norway, and data will be analysed in both countries. When results from the study are published, no information can be traced back to you or any other individual patient. You can say yes or no without giving any reasons. If you say no, you risk nothing and you will start treatment when you and your doctor think it is best. Everybody who is asked to particpate in the project will receive the same subsidising of their treatment during the first two years of treatment, whether they say yes or no. If you say yes, we ask you to put your signature at the consent formula at the bottom of this sheet. The project has been cleared by the Head of the Provincial Health Service in Ngaoundere and by the Regional Committee for Medical Research Ethics in Western Norway.

--------------------------------------------------------------------------------------------------------------

I consent to participate in the research project described to me. I understand that I can withdraw at any time, without giving any reasons.

___________________ (in writing or thumbprint)
